# Nerves and Irregular Hours of Sleep

**Published:** 1886-04

**Authors:** Benjamin W. Richardson


### NERVES AND IRREGULAR HOURS OF SLEEP.

BY BENJAMIN W. RICHARDSON, M. D.

The habit of keeping irregular hours of sleep, and of taking too little sleep, leads to serious forms of disease, and, indeed, I know of no habit which helps more surely to shorten life than that of fighting against natural periods of rest. I have seen the effects of this habit in members of my own profession, who too often set up a new world of their own when the rest of the world is in sleep; in politicians; in scholars, who habitually incline to work through the night; and in many more who are obliged by their occupation to watch while others sleep. In all these classes I have seen nothing but universal evil from the habit, imposed or self-imposed, of broken rest. In this observation I do not want rigidly to maintain that sleep must necessarily be taken at certain particular hours. I believe it to be best to take it at certain hours, including the first hours of the night, but I am now describing the effects following the habit of sleeplessness, in season and out of season, and the insomnia which is generated by such habit. In persons of vigorous constitution the habit of disregarding proper sleep, and the insomnia which springs from it, may go on for several years without any apparent bad effect. In time, however, it is certain to produce its natural consequences. The first indications of danger are irritability of mind and feverish excitement, followed by depression, pallor, and deficiency of appetite. These are succeeded by fits of unconsciousness, in which the affected person positively sleeps, and, it may be, sleeps soundly, without himself knowing the fact. In this way he gets rest, which for a little while may give a certain measure of relief; but soon the nervous failure increases, and one of two results succeeds. He either falls into a sleep which becomes a coma, and terminates in death, or he continues sleepless, unless artificially made to sleep by narcotics, and with progressing failing powers sinks into paralysis, to succumb from that affection. In exceptional cases the insomniac makes a fair recovery. Under regulated mode of life, and especially under the regulation which leads the sufferer to go to bed at unusually early hours, such as eight or nine o'clock, whether he can sleep or not at first, the insomia, or sleeplessness, is often cured without any artificial aid. It is, however, apt to return after mental strain or worry, and indeed may be expected always to return if the strain or worry be severe or prolonged,—*Field of Disease.*

**The** Empirics were the opponents of Dogmatist, the founder being Philenus, of Cos.

